# JUST THE TWO OF US? THE PSYCHOLOGICAL IMPACT OF MARITAL DISSOLUTION ON CHILDLESS OLDER ADULTS

**DOI:** 10.1093/geroni/igad104.0308

**Published:** 2023-12-21

**Authors:** Deborah Carr, Shinae Choi

**Affiliations:** Boston University, Boston, Massachusetts, United States; The University of Alabama, Tuscaloosa, Alabama, United States

## Abstract

An estimated 15% of U.S. adults ages 55+ are childless, with this proportion projected to increase among future cohorts. Of the 15 million childless older adults today, 40 percent are married – challenging the notion that childlessness is the domain of never married persons only. Little is known about the experiences of childless older adults who become widowed or divorced, and whether they suffer poorer mental health than their peers with children. Widowed and divorced persons lack the support of children yet may possess other resources or strengths as they age on their own. We use data from the 2016 and 2018 waves of the Health and Retirement Study (N = 16244) to evaluate whether parental status (childless, any living biological child(ren), stepchild(ren) only, all child(ren) now deceased) moderates the association between marital status (married, cohabiting, divorced/separated, widowed) and mental health (depressive symptoms, and social and emotional loneliness) and whether these patterns differ by gender. Multivariate moderation analyses show that childless widowed men and divorced men who lost a child to death have dramatically higher levels of social loneliness, relative to other men. Childlessness does not moderate the effect of marital status on loneliness or depressive symptoms among women. However, widowed and divorced women with stepchildren only evidence significantly higher depression and emotional loneliness. We discuss implications for understanding late-life mental health, underscoring that childlessness does not uniformly compromise mental health among unmarried persons, and that some parent-child relations (especially step-relations) may be a source of stress rather than solace.

